# Temperature Effects in Conventional and RAFT Photopolymerization

**DOI:** 10.1021/acs.macromol.4c02001

**Published:** 2024-12-23

**Authors:** Tochukwu Nwoko, Bo Zhang, Taylor Vargo, Tanja Junkers, Dominik Konkolewicz

**Affiliations:** † Department of Chemistry and Biochemistry, Miami University, 651 E High St, Oxford, Ohio 45056, United States; ‡ Polymer Reaction Design (PRD) Group, School of Chemistry, Monash University, 17 Rainforest Walk, Clayton VIC, Melbourne 3800, Australia

## Abstract

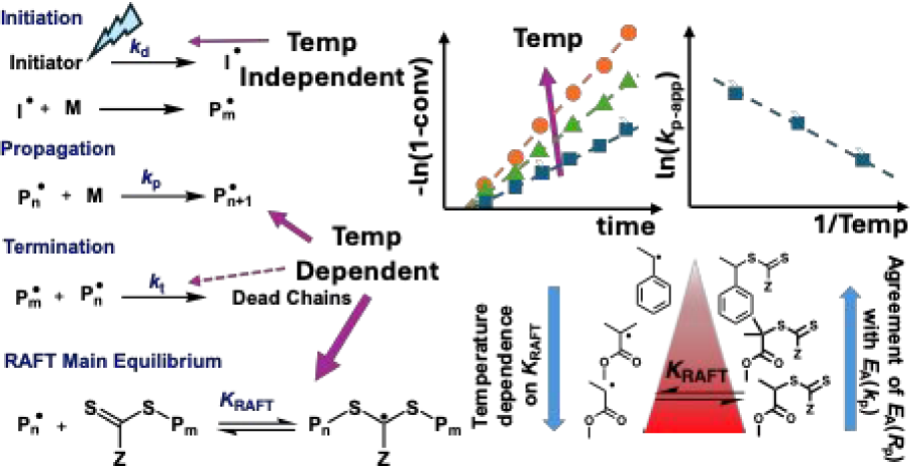

Photochemical processes are often thought to be temperature-independent. However, photochemical polymerization involves photochemical processes such as light-driven radical generation coupled with thermal-driven reactions such as monomer propagation. The apparent activation energy of propagation, *E*_A_(*R*_p_), of a series of three monomers, methyl acrylate (MA), methyl methacrylate (MMA), and styrene (STY), are deduced from Arrhenius analysis of conventional and RAFT photopolymerization of these monomers across a range of corresponding temperatures. The deduced *E*_A_(*R*_p_) was compared with the benchmarked *E*_A_(*k*_p_) derived from pulse laser polymerizations coupled with size exclusion chromatography (PLP-SEC). For conventional photopolymerization of MA, MMA and STY, the relatively small discrepancy between the photopolymerization-derived *E*_A_(*R*_p_) and the *E*_A_(*k*_p_) from PLP-SEC was rationalized due to temperature-induced changes in termination. The deviation between the *E*_A_(*R*_p_) measured in RAFT photopolymerization and *E*_A_(*k*_p_) from PLP-SEC depends on the retardation strength in RAFT polymerizations. MMA and STY monomers are characterized with minimal retardation and recorded excellent agreement in PLP-SEC and RAFT-derived *E*_p_ values. However, the RAFT photopolymerization of MA, which is subject to strong retardation, had a much larger *E*_A_(*R*_p_) than the *E*_A_(*k*_p_) from PLP-SEC. The high apparent *E*_A_(*R*_p_) in RAFT polymerization of MA is likely due to the added influence of temperature-induced changes in the RAFT equilibrium. Overall, these results rationalize temperature-dependent effects in photochemical reactions.

## Introduction

**Scheme 1:**
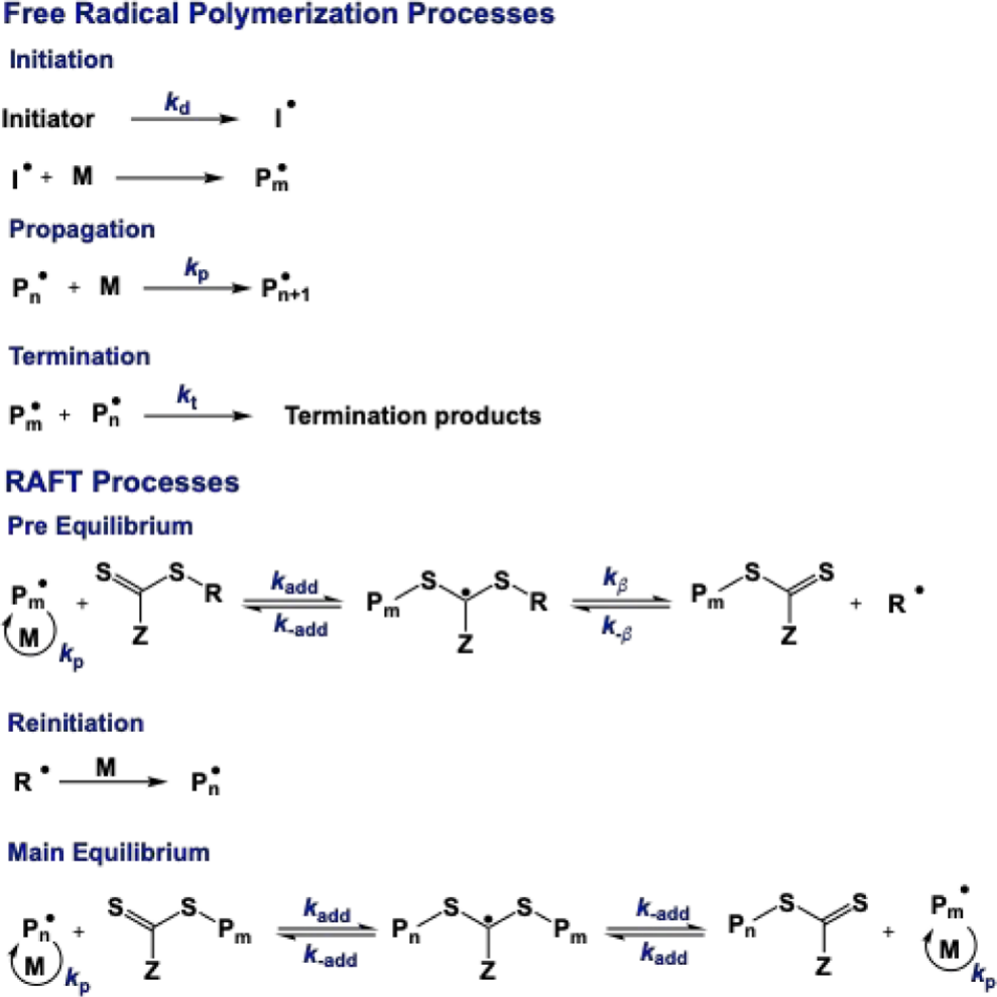
Mechanism of FRP and RAFT Polymerization

Conventional free radical polymerization (FRP) is the basis for reversible deactivation radical polymerization techniques, including reversible addition–fragmentation chain transfer (RAFT), atom transfer radical polymerization (ATRP), and nitroxide-mediated polymerization (NMP).^1,2^ Radical polymerization is compatible with a wide variety of polymer building units, allowing the synthesis of high molecular weight polymers and multiblock copolymers and offering compatibility with a broad range of monomers.^2,3^ Radical polymerization can be performed under mild reaction conditions, offering tolerance to impurities and functional groups and the ability to be performed in aqueous media and across a wide range of temperatures.^4^ The broad scope of the processes has led to the adoption of both RAFT and FRP in both commercial and laboratory-scale polymer syntheses.^5^ To ensure efficiency and predictability in these polymerization techniques, extensive research efforts have been applied toward understanding and optimizing the kinetics and mechanisms of radical polymerizations. Both ideal FRP and RAFT processes contain three core mechanistic steps: initiation, propagation, and termination,^6^ with RAFT having an additional degenerative transfer process that enables control over the polymer microstructure.^7^ The initiation steps are a combination of both initiator decomposition and first monomer addition, which yields a growing macroradical. In the decomposition step, as seen in Scheme 1, the initiator generates radicals either by heat (thermal initiation) or by light (photoinitiation), yielding primary radicals.

Most radical polymerizations use heat for initiation. However, light driven or photoinitiation is also possible. Photochemical radical initiators, compared to thermal initiators, have several advantages including that photoinitiator decomposition rates are not a function of temperature, but rather of the intensity of incident radiation.^8^ Therefore, photoinitiation allow the radical generation processes to be decoupled from temperature, with initiation processes controlled by where and when light sources are present, enabling spatiotemporal control over radical generation.^6^ Even though photopolymerization uses light to generate radicals, the subsequent propagation, termination or RAFT exchange processes are conventional thermally controlled reactions, hence they will have conventional temperature dependencies given by Arrhenius relationships in the rate coefficients.^9,10^

In idealized FRP, the steady state radical concentration is given by eq 1 by balancing the rates of initiation and termination, where *k*_d_ is the rate coefficient for initiation, *k*_t_ is the rate coefficient for termination, *f* is the radical initiator efficiency and [I] is the initiator concentration.^11^ The steady state radical concentration of eq 1 can be combined with the rate coefficient for propagation, *k*_p_, and monomer concentration, [M], to give the ideal FRP propagation rate *R*_p_ in eq 2.^11^ Purely photochemical reactions are essentially temperature independent. This is due to the very high energy present in photons, therefore the photochemical decomposition of an initiator to 2 free radicals should have essentially no temperature dependence. Since the *k*_p_ and *k*_t_ follow an Arrhenius relationship,^9^ coupled with the near temperature independence of photochemical initiation, the activation energy, gives the apparent activation energy for the rate of photopolymerization can be estimated by eq 3.
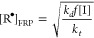
1
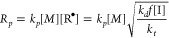
2
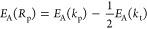
3

In eq 3, *E*_A_(*R*_P_) is the apparent activation energy of photopolymerization. *E*_A_(*k*_P_) is the activation energy of propagation which is well characterized by pulsed laser polymerization coupled with size exclusion chromatography (PLP-SEC) for many monomers including methyl methacrylate (MMA), styrene (STY), and methyl acrylate (MA).^9^*E*_A_(*k*_t_) is the activation energy of termination and varies slightly between monomers such as MMA, STY, and MA. *E*_A_(*k*_t_) is affected by the solvent, but is often found to be around 10 kJ/mol or lower.^9^ It should be noted that in thermal polymerization, eq 3, would need to include the activation energy of the thermal initiator decomposition, a contribution that high, typically over 100 kJ/mol,^12^ hence having a distinct effect on *E*_A_(*R*_P_)·

In recent decades there has been a growing interest in controlling the structure of polymers made by radical polymerization and photopolymerization using methods such as RAFT.^13,14^ In RAFT, the propagating radical undergoes a reversible addition reaction to the chain transfer agent (CTA) to form an intermediate radical that subsequently fragments, yielding a macroRAFT species and a new propagating radical,^2,14^ establishing the chain length controlling equilibrium. If fragmentation occurs fast enough, the CTA should not impact the polymerization rate significantly, and in principle, the average radical concentration in conventional free radical polymerization, [R**^•^**]_FRP_ should be comparable to a RAFT polymerization system, [R**^•^**]_RAFT_, where [R**^•^**]_FRP_ is given in eq 1.^15 −17^ However, prior work indicates significant retardation in polymerization rates in RAFT systems compared to their FRP counterparts across many monomer-CTA pairs.^16^ In these polymerization systems, a consistent reduction in polymerization rate was observed with increasing CTA concentrations. A scaling law was derived that predicts a relationship between [R**^•^**]_FRP_ in free radical polymerization and [R**^•^**]_RAFT_ in RAFT polymerization systems^17^ as a function of the RAFT equilibrium constant, *K*_RAFT_ and the CTA concentration as shown in the eq 4, with overall polymerization rate given by eq 5, assuming intermediate radical termination.
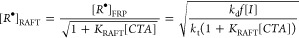
4
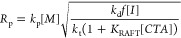
5

For strongly retarding monomer-CTA pairs, the product of *K*_RAFT_ × [CTA] is substantially greater than 1, and in less retarding monomer systems, substantially less than 1. This indicates that for strongly retarding monomer-CTA pairs, any temperature dependence in the RAFT equilibrium constant will also impact the overall temperature dependence of RAFT photopolymerization in addition to the earlier effects on *k*_p_, and *k*_t_. Thus, by probing the differences in activation energies of the rate of polymerization between RAFT and FRP, one can deduce information about the RAFT equilibrium constant with respect to its temperature dependence. Due to the simplicity of eq 3, photopolymerization is particularly suited for such a comparison because the activation energy of propagation is well-known, and no initiation activation energy needs to be considered.

This work explores the effect of temperature on the overall polymerization rate coefficient of photopolymerization systems. Unlike prior work, which shows a phenomenological change in the polymerization rate with temperature in photopolymerization,^18^ here we seek a quantitative comparison of temperature effects in radical photopolymerization with the underlying temperature effects in other thermal reactions such as the propagation process or RAFT exchange in strongly retarded systems. Temperature effects were first studied using conventional radical polymerization of methyl methacrylate (MMA), styrene (STY), and methyl acrylate (MA) in continuous flow. Subsequently, MMA, STY, and MA were subjected to RAFT photoinitiated polymerization with the underlying apparent activation energy connected to the temperature dependence of propagation and RAFT exchange where applicable.

## Results and Discussion

**Figure 1:**
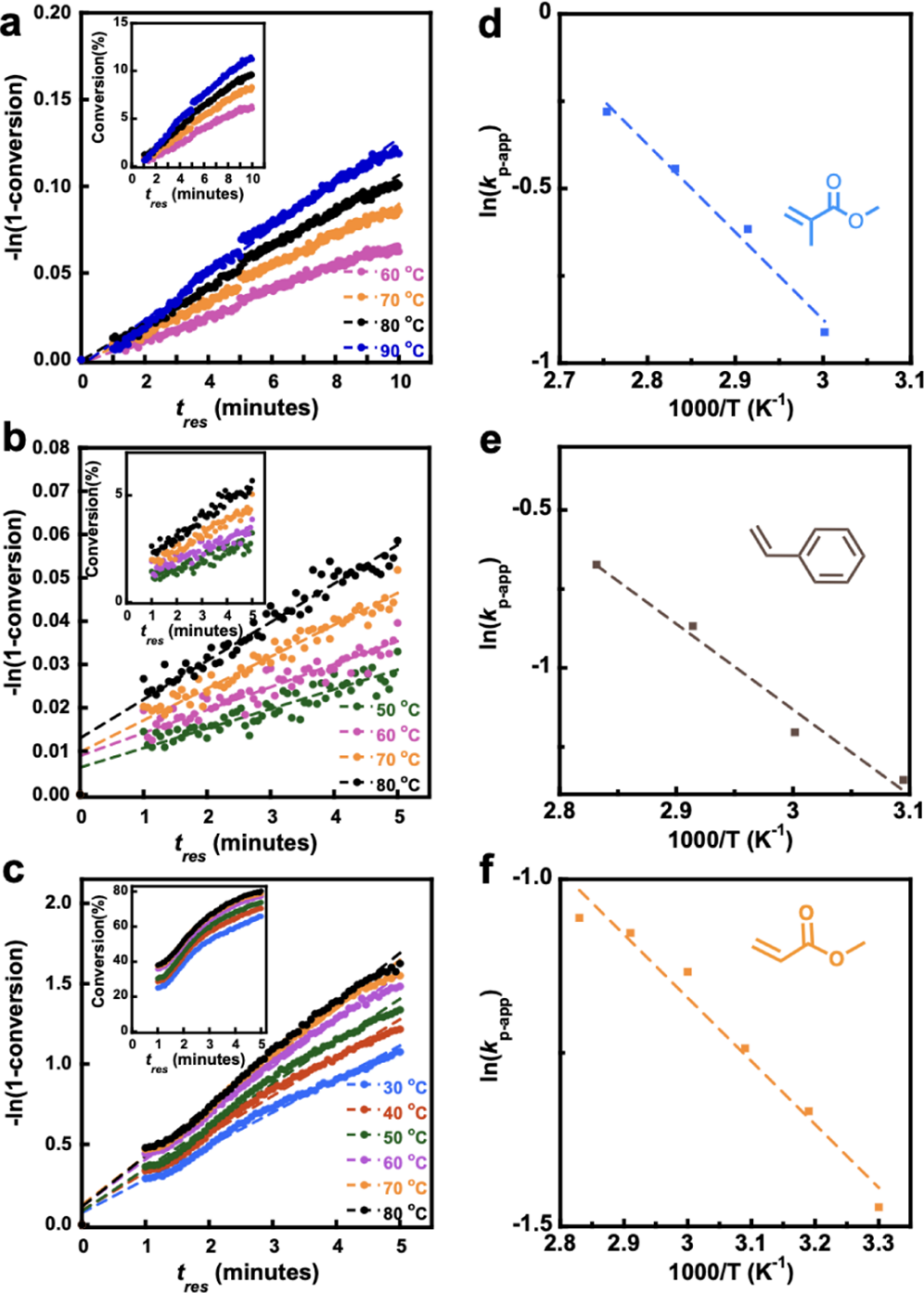
Time-sweep data for continuous flow polymerization of MMA, STY, and MA at each temperature. First order plot of with inset showing raw conversion vs residence time for photopolymerization of (a) MMA, (b) STY, and (c) MA. Arrhenius analysis of apparent rate propagation rate coefficient, *k*_p-app_, as deduced from first order kinetics in for (d) MMA, (e) STY, and (f) MA.

Initially, the temperature dependence of photochemically initiated conventional radical polymerization of MMA, STY and MA in continuous flow was measured. Each monomer was polymerized in dimethylformamide (DMF) under blue light (450 ± 10 nm and 5.3 ± 0.2 mW/cm^2^)^8^ using 1-dodecanethiol as an irreversible chain transfer agent and phenylbis(2,4,6-trimethylbenzoyl)phosphine oxide (BAPO) as the photoinitiator. A conventional CTA was required at low concentrations to limit the viscosity of solutions and to provide conditions under which the reactor would behave ideally. Temperature effects in this conventional radical photopolymerization were determined by following the kinetics of polymerization at temperatures between 60 and 90 °C for MMA, 50 and 80 °C for STY, 30 and 80 °C for MA, via transient continuous flow timesweep experiments (for details on the methodology of such experiments see reference [^19^]). As seen in Figure 1 in all cases, a consistent increase in polymerization rate with increasing temperature was recorded. This allowed Arrhenius analysis to extract the apparent activation energy of the polymerization, *E*_A_(*R*_p_). This can be derived by analyzing the pseudo-first-order kinetic plots in Figure 1a-c, noting that the slope of the semilogarithmic or first-order plot is *k*_p-app_, which is related to the propagating radical concentration ([R^•^]) and *k*_p_ as follows:
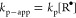
6

In all cases a close to linear first order kinetic plot was measured, with deviations from linearity potentially arising from initial temperature ramping and possibly depletion of the radical photoinitiator. From the flow process in Figure 1, experimental values of *E*_A_(*R*_p_) were determined. These values were *E*_A_(*R*_p_) = 21 ± 2 kJ/mol for MMA photopolymerization *E*_A_(*R*_p_) = 23 ± 3 kJ/mol for STY photopolymerization, and *E*_A_(*R*_p_) = 7.6 ± 0.7 kJ/mol for MA photopolymerization. It should be noted that due to the nature of the flow online monitoring, no access is given to low reaction times.^19^ This does, however, not negatively impact the evaluation since the first order plot is linear, and because back-extrapolation of the measured data to the coordinate origin shows a good match with expectations. For the linearity of the plots, one might argue that they show a slight curvature. This stems from nonideal plug flow conditions and the underlying residence time distribution present in the reactor. Yet, this deviation from the ideally fully linear behavior is very small and practically negligible compared to the typical scatter in a batch experiment. In each case, the determined activation energies are lower than the *E*_A_(*k*_p_) derived from PLP-SEC of *E*_A_(*k*_p_) = 22.4 ± 0.3 kJ/mol for MMA,^20^*E*_A_(*k*_p_) = 32.5 kJ/mol for STY,^21^ and *E*_A_(*k*_p_) = 17.3 ± 0.3 kJ/mol for MA.^22^ However, radical termination may account for the disparity in activation energies between the photopolymerization (*E*_A_(*R*_p_)) and propagation as measured by PLP-SEC (*E*_A_(*k*_p_)), as highlighted in eq 3. For MMA eq 3 using the parameters *E*_A_(*k*_p_) = 22.4 ± 0.3 kJ/mol and *E*_A_(*k*_t_) = 5.6 ± 2.6 kJ/mol,^9^ gives a predicted *E*_A_(*R*_p_) = 20 ± 2 kJ/mol. Similarly, for STY eq 3 with the values *E*_A_(*k*_p_) = 32.5 kJ/mol and *E*_A_(*k*_t_) = 8.9 ± 3.2 kJ/mol^9^ gives a predicted *E*_A_(*R*_p_) = 28 ± 2 kJ/mol. Finally, MA using eq 3 and *E*_A_(*k*_p_) = 17.3 ± 0.3 kJ/mol and *E*_A_(*k*_t_) = 9 ± 6 kJ/mol,^9^ gives a predicted *E*_A_(*R*_p_) = 12 ± 3 kJ/mol.

Considering the typical activation energies for thermally induced FRPs, the discrepancy seen here is almost negligible and the result is in acceptable agreement between the experimentally measured value of *E*_A_(*R*_p_) and the value of *E*_A_(*R*_p_) predicted by eq 3, especially given that an ideal radical polymerization scheme is assumed for the calculation above. These results are summarized in Table 1.

**Table 1:** Summary of Estimated *E*_A_(*R*_p_) Values in All the Monomer Systems, Including Their Predicted Valued Compared to PLP-SEC Derived *E*_A_(*k*_p_) and *E*_A_(*k*_t_)

**Monomer**	*E*_A_(*k*_p_) PLP-SEC (kJ/mol)	*E*_A_(*k*_t_) PLP-SEC (kJ/mol)	*E*_A_(*R*_p_) FRP (kJ/mol) Expt	*E*_A_(*R*_p_) FRP (kJ/mol) Pred	*E*_A_(*R*_p_) RAFT (kJ/mol) Expt	*E*_A_(*R*_p_) RAFT (kJ/mol) Pred
MA	17.3 ± 0.3^22^	9 ± 6^9^	7.6±0.7	12 ± 3^a^	47 ± 4	60 ± 8^b^
MMA	22.4 ± 0.3^20^	5.6 ± 2.6^9^	21 ± 2	20 ± 2^a^	25.0 ± 0.4	20 ± 2^a^
STY	32.5^21^	8.9 ± 3.2^9^	23 ± 3	28 ± 2^a^	37.3± 0.8	28 ± 2^a^

a Predicted value of *E*_A_(*R*_p_) calculated using eq 3.

b Predicted value of *E*_A_(*R*_p_) calculated using eq 7.

**Scheme 2:**
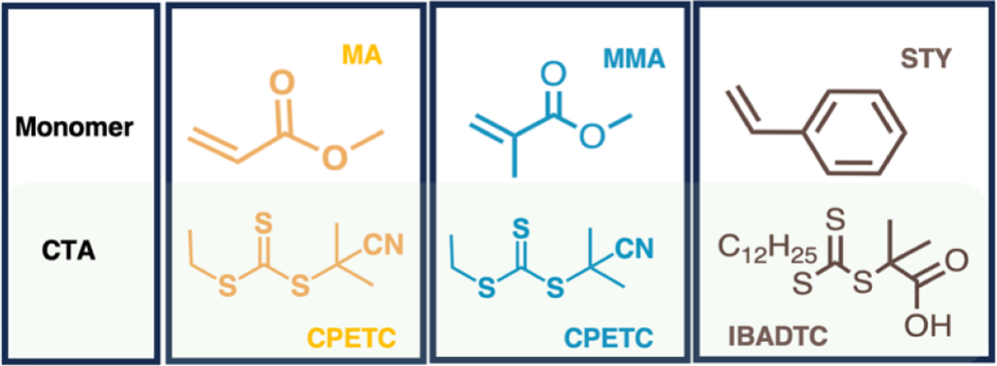
Monomers and CTA Explored in for the Photopolymerization Temperature-Dependence Investigation

Having established the temperature dependence of FRP, the impact of temperature on RAFT photopolymerization was determined on the same three more active monomers (MAMs), MMA, STY, and MA. All polymerization systems were photoinitiated with BAPO as the photoinitiator. 2-Cyano-2-propyl ethyl trithiocarbonate, (CPETC) was used as the CTA in MA and MMA and IBADTC as the chain transfer agent (CTA) in STY, based on successful literature precedent^14,19^as highlighted in Scheme 2. Different temperature ranges were applied to the monomer systems for Arrhenius analysis. For MA, the covered temperature range is 25–55 °C, for MMA it is 45–65 °C, and for STY it is 35–65 °C. All sample vials were exposed to blue light radiation at 450 ± 10 nm and 12.7 ± 0.6 mW/cm^28^·

**Figure 2:**
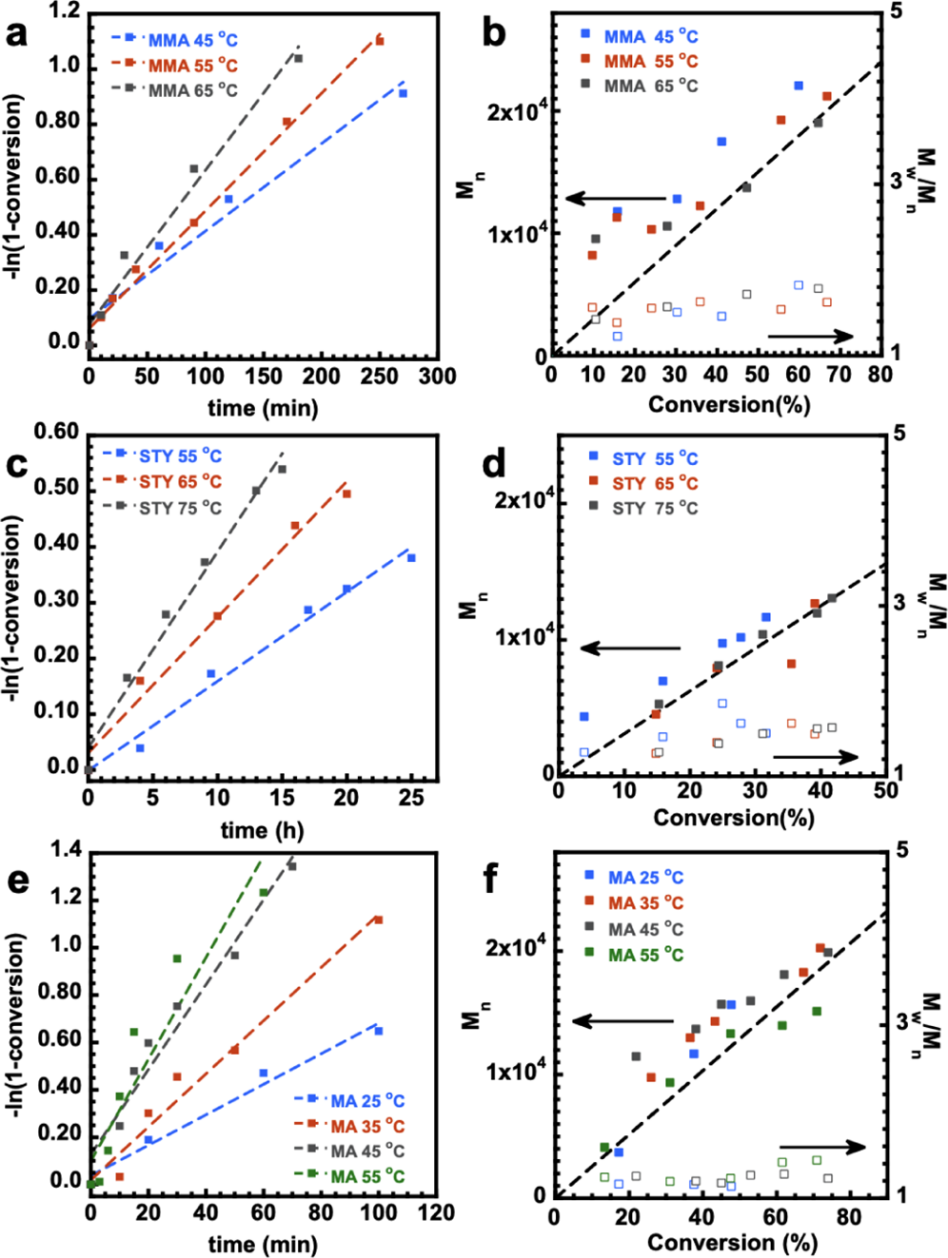
Kinetics and evolution of *M*_n_ (solid points) and *M*_w_/*M*_n_ (hollow points) with conversion and kinetic plots for each temperature setting in the RAFT polymerization of MMA, STY and MA. (a) Kinetics of RAFT photopolymerization of MMA, (b) evolution of *M*_n_ and *M*_w_/*M*_n_ with conversion in RAFT photopolymerization of MMA, (c) kinetics of RAFT photopolymerization of STY, (d) evolution of *M*_n_ and *M*_w_/*M*_n_ with conversion in RAFT photopolymerization of STY, (e) kinetics of RAFT photopolymerization of MA, and (f) evolution of *M*_n_ and *M*_w_/*M*_n_ with conversion in RAFT photopolymerization of MA.

**Figure 3:**
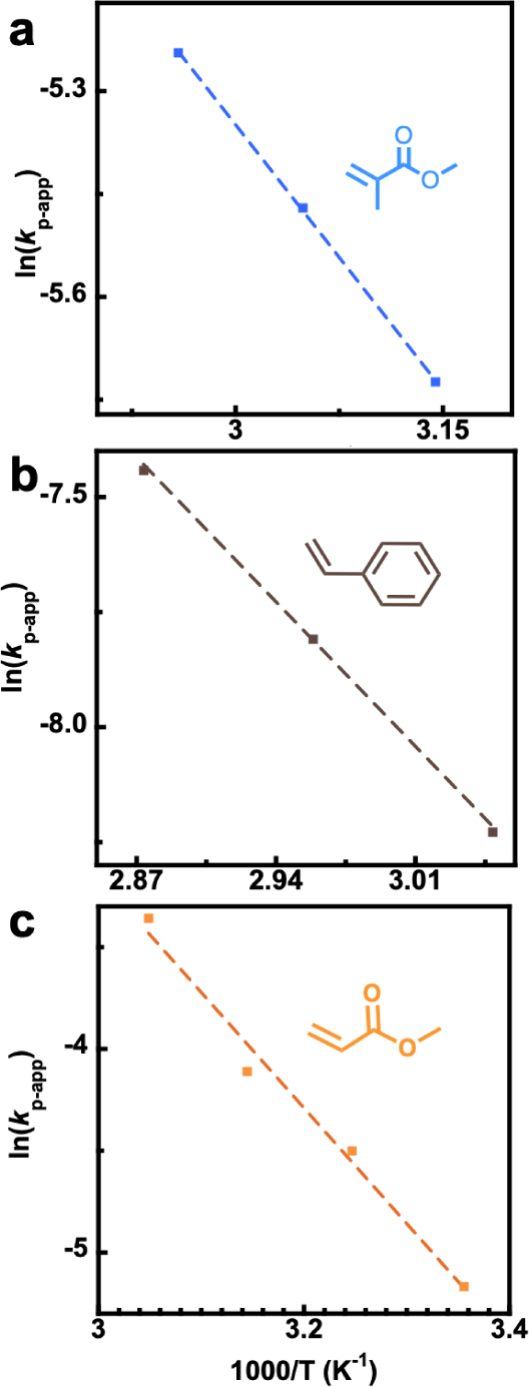
Arrhenius plots for (a) MMA, (b) STY and (c) MA photo-RAFT polymerizations.

Figure 2 depicts the kinetic data, the evolution of number averaged molecular weight (*M*_n_), and molar mass dispersity (*M*_w_/*M*_n_) for the polymerization of MA, MMA, and STY in dimethylformamide (DMF) at the respective temperature ranges and CTA choice. It is notable that the control experiment including all reaction components was set up for MA and MMA. In these controls, no BAPO was added, and the other reaction conditions were unchanged. These control experiments with no BAPO showed essentially no polymerization, ruling out the possibility of substantial radical generation through photoiniferter processes. In all cases with BAPO, higher temperatures led to measurably higher polymerization rates. In all cases, there was almost no variation in the correlation of experimental *M*_n_ and theoretical *M*_n_ (*M*_n-th_) across the temperature studies. However, it appears that the STY polymerization exhibited higher dispersity at a lower temperature of 55 °C, while the MMA and MA systems showed negligible effect of temperature on dispersity. For all monomers, *E*_A_(*R*_p_) was deduced through Arrhenius analysis and compared with PLP-SEC derived *E*_A_(*k*_p_) as seen in Table 1. All Arrhenius analyses of the RAFT systems can be found in Figure 3.^19^

MMA and STY have *E*_A_(*R*_p_) values measured by temperature_–_dependent photopolymerization that are similar to *E*_A_(*k*_p_) measured by PLP-SEC. For MMA, *E*_A_(*k*_p_) = 22.4 kJ/mol^20^ and *E*_A_(*R*_p_) = 25.0 ± 0.4 kJ/mol by PLP-SEC and in RAFT photoinitiation respectively. Similarly for STY, the *E*_p_ values are 32.5 kJ/mol^21^ and 37.3 ± 0.8 kJ/mol by PLP-SEC and in RAFT photoinitiation respectively. Factoring in the activation energies of termination, *E*_A_(*k*_t_), which are *E*_A_(*k*_t_) = 5.6 ± 2.6 kJ/mol for MMA and *E*_A_(*k*_t_) = 8.9 ± 3.2 kJ/mol for STY,^9^ allows an estimate of the predicted activation energy for photopolymerization. Unfortunately, accounting for termination shifts the predicted activation energies slightly away from the measured values. For styrene eq 3 estimates *E*_A_(*R*_p_) = 28 ± 2 kJ/mol (measured value of *E*_A_(*R*_p_) = 37.3 ± 0.8 kJ/mol) and for MMA 20 ± 2 kJ/mol, (measured value of *E*_A_(*R*_p_) = 25.0 ± 0.4 kJ/mol). This result can again be seen as a reasonable match between experiments. Like in the case of the thiol-mediated FRP in flow, numbers indicate larger complexities of a radical polymerization compared to the ideal polymerization scheme. Nonetheless, the difference is relatively small given the general difficulties (and hence the scarcity of availability) in determining activation energies for the termination reaction.

In contrast, MA showed a substantially larger discrepancy between RAFT photopolymerization and PLP-SEC, which cannot be attributed to the smaller effects as discussed above. For MA, PLP-SEC determined *E*_A_(*k*_p_) to be 17.3 kJ/mol.^22^ Using eq 3 with *E*_A_(*k*_t_) = 9 ± 6 kJ/mol for MA,^9^ gives the same predicted *E*_A_(*R*_p_) = 12 ± 3 as in the conventional radical polymerization system. Yet, *E*_A_(*R*_p_) is determined to 47 ± 4 kJ/mol for the RAFT photopolymerization, which is in poor agreement when comparing the experiment with theory using eq 3 alone. A further explanation for this significant gap of 30 kJ/mol needs to be identified.

In previous work, MAMs were subjected to RAFT polymerization. A scaling law as seen in eq 4 simplified the retardation analysis across monomer systems. This scaling analysis revealed that MMA and STY display relatively weak retardation in RAFT polymerization. Based on prior work under typical polymerization conditions (*K*_RAFT_ × [CTA] < 1)^16^ MMA and STY displayed minimal retardation with trithiocarbonate CTAs.^17^ In these cases, there is negligible impact of the CTA on the concentration of propagating radicals in free radical polymerization and RAFT polymerization. Hence in both RAFT and conventional radical polymerizations of MMA and STY, this is reflected in the similar values of *E*_A_(*R*_p_) determined by the RAFT photopolymerization and *E*_A_(*R*_p_) based on eq 3. However, in prior studies, trithiocarbonate mediated RAFT polymerization of MA displayed strong retardation effects,^16,17^ as seen by substantial reductions in polymerization rate with increasing the CTA concentrations. Due to the strong retardation effects in trithiocarbonate mediated MA photopolymerization, the rate in RAFT polymerization is expected to be dissimilar to that in conventional radical polymerization. This is because for MA polymerization mediated by trithiocarbonate CTAs, *K*_RAFT_ × [CTA] ≫ 1 under typical polymerization conditions.^16,17^ Therefore, both the temperature dependence of the RAFT equilibrium constant, *K*_RAFT_, and the temperature dependence of *k*_p_ and *k*_t_ need to be considered in the overall apparent *E*_p_ in RAFT photopolymerization. As highlighted in the SI Van’t Hoff type analysis in *K*_RAFT_ (), in addition to the PLP-SEC derived activation energy for propagation and termination can be combined. As derived in the SI, *E*_A_(*R*_p_) for a strongly retarded RAFT polymerization is predicted by
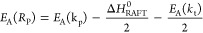
7

Experiments using dithiobenzoate mediated RAFT polymerization of butyl acrylate suggest that the RAFT equilibrium constant is strongly temperature dependent, decreasing significantly as the temperature increases.^10^ Unfortunately, enthalpy changes and temperature dependencies of the RAFT equilibrium constant are not experimentally available for acrylate/trithiocarbonate systems to the best of our knowledge. However, computational work from Coote et al. indicate a  = −95 kJ/mol for acrylate trithiocarbonate systems,^23^ with typical errors in quantum calculations being in the order of 10–14 kJ/mol as determined in a broad benchmarking study.^24^ Substituting the *E*_A_(*k*_p_) from PLP-SEC of 17.3 kJ/mol, *E*_A_(*k*_t_) = 9 ± 6 kJ/mol^24^ and using an estimated enthalpy for *K*_RAFT_ of  = −95 ± 14 kJ/mol for acrylate monomers and trithiocarbonate in eq 7, gives^24,25^ a predicted *E*_A_(*R*_p_) = 60 ± 8 kJ/mol which is much closer to the 47 ± 4 kJ/mol measured herein.

It should be noted that the obtained value is in line with previous observations on the overall size of the RAFT equilibrium constant. Fragmentation of the RAFT intermediate is a beta-scission reaction, which is typically associated with a high activation energy, leading to the large offset observed here for *E*_A_(*R*_p_). Thus, the study of the temperature dependence of photoinitiated RAFT reactions offers the opportunity to qualitatively estimate the relative size of the equilibrium constant, which is interesting given the uncertainties that persist for this quantity.^26^ Termination of the RAFT intermediate, which typically presents challenges in *K*_RAFT_ determination, may play a smaller role in photoinitiated RAFT for similar reasons to those that cause conventional termination to have only a small impact on the temperature dependence of FRP.

## Conclusions

The temperature dependence of radical photopolymerizations was found to be primarily explained by the activation energy of the propagation rate coefficient *k*_p_. A key conclusion is that the kinetics and rate of radical photopolymerizations are in fact temperature dependent. While this is consistent with the predictions of polymerization kinetics, it is noteworthy because a general temperature independence is sometimes assumed, neglecting the temperature activation of the propagation reaction. Pronounced temperature effects are often identified for radical photopolymerizations, which have been quantified in this study. Assuming ideal polymerization conditions, the identified activation energy for the overall rate of polymerization in a conventional thiol-mediated radical polymerization is found to be close to the activation energy of the propagation rate coefficient, factoring in corrections due to termination. The same is true for the RAFT photopolymerization under blue light for MMA and STY. However, a significantly larger gap between the activation energy for the overall rate of polymerization and the activation energy of the propagation rate coefficient was found for the photoinitiated RAFT polymerization of MA. This discrepancy can be correlated with the retarding nature of the CTA/monomer system. In the case of a strongly retarding RAFT system of MA, the temperature dependence of photopolymerization is given by the temperature dependence of the RAFT equilibrium, in addition to propagation and termination. Overall, this study quantifies the temperature dependence of photoinitiated FRP and RDRP processes and identifies the sources of the observed temperature dependencies. This work highlights the potential benefits of using temperature in conjunction with photochemistry to drive efficient polymerization processes.

## Supporting Information Available

Experimental details, calibration data of the flow reactor, derivation of temperature dependence in RAFT polymerization under strong retardation, statistical analysis of Arrhenius plots (PDF)
