# Supplementary material for: Temperature Effects in Conventional and RAFT Photopolymerization
Source: Macromolecules. 2024 Dec 23;58(1):488–94. doi: 10.1021/acs.macromol.4c02001 (PMC11740896; doi:10.1021/acs.macromol.4c02001)
Supplement: Supplementary file 1 — ma4c02001_si_001.pdf [file ma4c02001_si_001.pdf]

Supporting Information: Temperature Effects in Conventional and RAFT Photopolymerization

Tochukwu Nwoko[a], Bo Zhang,[b] Taylor Vargo[a], Tanja Junkers,[b] Dominik Konkolewicz[a, *]

[*]Correspondence: d.konkolewicz@miamiOH.edu

[a] Department of Chemistry and Biochemistry, Miami University, 651 E High St. Oxford, OH, 45056, USA

[b] Polymer Reaction Design (PRD) Group, School of Chemistry, Monash University, VIC, 3800, Australia

Experimental

1. Materials

All materials were purchased from commercial sources and used as received unless otherwise stated. The chain transfer agents, 2-cyano-2-propyl ethyl trithiocarbonate (CPETC)[1] and 2-[[(dodecylthio)thioxomethyl]thio]-2-methylpropanoic acid (IBADTC)[2] were prepared from known methods.

2. Characterization

NMR

All Nuclear magnetic resonance was performed on a Bruker 400 MHz spectrometer unless otherwise specified to be on a Bruker 500 MHz spectrometer.

Size Exclusion Chromatography (SEC)

All experimental molecular weights of polymers of MMA, STY, MA were determined by size exclusion chromatography (SEC), using poly(methyl methacrylate) standards. The SEC system

was an Agilent SEC system equipped with an autosampler, an Agilent 1260 isocratic pump, 1× Agilent MixedB-guard and 2× Agilent Mixed-B analytical columns and an Agilent 1260 refractive index (RI) detector. The eluent was tetrahydrofuran (THF) at 30 °C with a flow rate of 1mL/min. The system was calibrated with poly (methyl methacrylate) standards with molecular weights in the range of 617500 to 1010. All samples were filtered through a 200nm PTFE filter prior to injection. Theoretical molecular weights were calculated as $M_{n,\ Theory} = M_{monomer} \times [monomer]_o/([CTA]_o) \times$ Monomer Conversion, where $M_{monomer}$ is the molecular weight of monomer, $[monomer]_o$ is the initial concentration, $[CTA]_o$ is the initial CTA concentration.

Fourier transform infrared (FT-IR)

ReactIR (Mettler Toledo) with a 50 ul DS micro flow cell was used to real-time inline monitor the concentration of monomer. The equipment was operated by the ICIR software, and the sample interval was 5 seconds, after finishing every scan it automatically generate a CSV file, containing the raw data of the IR spectrum

For the polymerization of MA, MMA and Sty the absorption peak of the C=C stretching at around 1292 - 1276, 1313 – 1288 and 884-947 cm^{-1} was monitored,respectively[3]. Firstly, polymerizations with varying monomer concentrations were carried out at room temperature to obtain the integral peak area of C=C stretching from IR spectra. Then, a calibration curve between the concentration of monomer and the integral peak area was built (Figure S1).

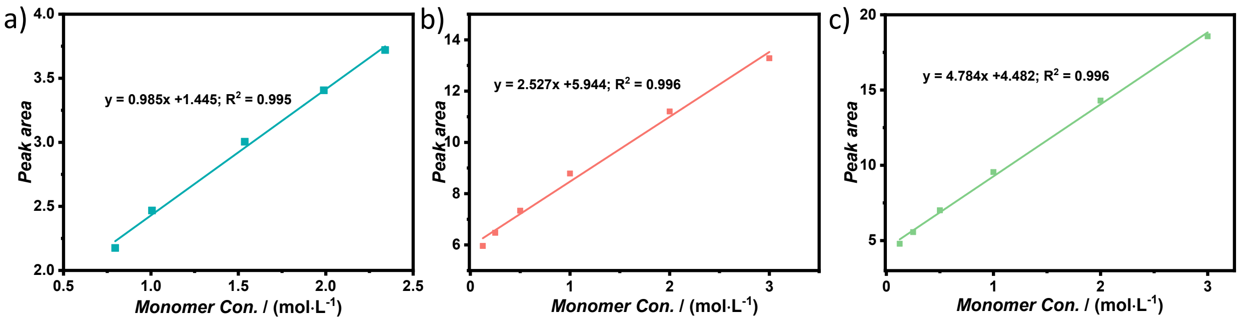

Figure S1. Calibration curve of a) MA, b) MMA, c) Sty concentration and integral peak area of the C=C stretching obtained from IR spectra.

3. Chain Transfer Synthesis Procedure

Synthesis of 2-cyano-2-propyl ethyl trithiocarbonate (CPETC)

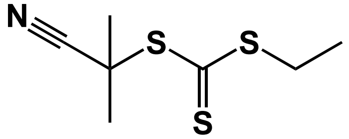

Ethanethiol (10.5 mL, 0.839 g/mL, 140.0 mmol) was added dropwise to a stirred solution of sodium hydroxide (6.14 g, 153.0 mmol) in 50 mL water at 0 °C. After 1 h, CS_2 (10 mL, 12.66 g, 166.0 mmol) was added dropwise to the reaction at 0 °C, and a yellow color was formed. The reaction was allowed to stir overnight. The reaction was washed with diethyl ether (3 × 80 mL) getting rid of the unreacted CS_2. To the aqueous layer, a solution of potassium ferricyanide in water was added until a yellow oil was formed. The yellow oily product was extracted with diethyl ether (3 × 80 mL) and concentrated under reduced pressure before being redissolved in ethyl acetate. AIBN (11.0 g, 66.9 mmol) was added to the resulting solution which was refluxed overnight at 70 °C. After 20 h, the reaction was cooled to room temperature and concentrated under reduced pressure. The residue was purified via flash chromatography (15 × 5.0 cm; 1:9 ethyl acetate/hexane) to afford CPETC as orange liquid (15.1 g, 52%). ^{1}H NMR (400 MHz, $CDCl_3$) d ppm: 1.35 (t, J = 7.4 Hz, 3H, $\underline{CH_3}CH_2$); 1.82 (s, 6H, $\underline{(CH_3)_2}CCN$); 3.33 (q, J = 7.4 Hz, 2H, $CH_3\underline{CH_2}S$) in agreement with the literature.[4]

Synthesis of (isobutricacid)yldodecyl trithiocarbonate (iBADTC)

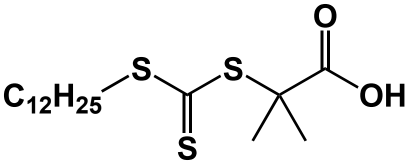

iBADTC was synthesized in accordance with the procedure reported in the literature[5]. Dodecanethiol (5.0 mL, 21 mmol, 14 eq) was dissolved in a suspension of K_3PO_4 (3.5 g, 16.5 mmol, 1.1 eq) in acetone (60 mL at ambient temperature) (4 mL water + 56 mL acetone) After stirring for 20 minutes at ambient temperature, carbon Sulfide was added (2.72 mL, 45 mmol, 3.0 eq) The reaction mixture was stirred additional 20 minutes at ambient temperature. 2-Bromo-2-methylpropionic acid (2.505 g, 15 mmol, 1.0 eq) was added and the reaction was stirred overnight

at ambient temperature. 1M HCl (200 mL) was added, and the mixture was extracted with DCM (2× 150 mL). The combined organic phases were washed with deionized water (75 mL), saturated aqueous brine solution (75 mL) and dried over anhydrous magnesium sulfate. The solvent was evaporated under reduced pressure. The crude product was purified by recrystallization from hexane to afford dodecylthiocarbonylthio-2-methylpropanoic acid (5.2 g 14.3 mmol, 95% recovery) as pale-yellow crystals. ^{1}H NMR (400 MHz, $CDCl_3$, δ) 3.28 (t, J = 7.6 Hz, 2H), 1.72 (s, 6H), 1.67 (t, J=7.1 Hz, 2H); 1.38 (m, 2H), 1.25 (br, 16H), 0.88 (t, J= 6.7 Hz, 3H)

4. Polymerization

Polymerization of Methyl methacrylate

In a 4 mL vial equipped with a Teflon stir bar was added Phenylbis(2,4,6-trimethylbenzoyl) phosphine oxide, BAPO (0.0043 mmol, 0.00067 equiv), CPETC (0.021 mmol, 0.0033 equiv) MMA (0.64 g, 0.68mL, 2.4 M) and 1.98 mL of DMF. Sample solution filled the vials to the second third while mineral oil was added to the brim to keep out oxygen. Sample vial was placed in water bath with temperatures 45, 55 and 65 °C. Separate sample vials were assigned to distinct time points of periods between 0 and 270 minutes. Sample vial distance from blue light source was 14 cm.

Polymerization of Styrene

In a 4 mL vial equipped with a Teflon stir bar was added Phenylbis(2,4,6-trimethylbenzoyl) phosphine oxide, BAPO (0.017 mmol, 0.0027 equiv), IBADTC (0.021mmol, 0.0033 equiv) STY (0.66 g, 0.73 mL, 2.36 M) and 2.2 mL of DMF. Sample solution filled the vials to the second third while mineral oil was added to the brim to keep out oxygen. Sample vial was placed in water bath with temperatures 55,65 and 75 °C. Separate sample vials were assigned to distinct time points of periods between 0 and 1500 minutes. Sample vial distance from blue light source was 14 cm.

Polymerization of Methyl Acrylate

In a 4 mL vial equipped with a Teflon stir bar was added Phenylbis(2,4,6-trimethylbenzoyl) phosphine oxide, BAPO (0.0044 mmol, 0.00067 equiv), CPETC (0.022 mmol, 0.0033 equiv) MA (0.57 g, 0.6 mL, 2.5M) and 2.0 mL of DMF. Sample solution filled the vials to the second third while mineral oil was added to the brim to keep out oxygen. Sample vial was placed in water bath with temperatures 25, 35, 45 and 55 °C. Separate sample vials were assigned to distinct time points of periods between 0 and 160 minutes. Sample vial distance from blue light source was 14 cm.

Free radical polymerization in continuous flow reactor

In a typical procedure, two stock solutions were prepared in volumetric flasks. One solution contains monomer (MA, or MMA or Sty) and 1-dodecanthiol (5 M and 0.009M respectively) in dimethylformamide (DMF). The second one contains BAPO (0.01 M) in DMF. Then stock solutions and pure solvent DMF were transferred to 20 mL glass vials sealed with rubber septa, and individually purged with argon for at least 5 minutes. Next, the solutions and pure solvent were transferred to three 10 mL gastight syringes (Trajan Scientific), preflushed with argon, and placed in the holder of syringe pumps. The concentration of stock solutions, reactor parameters (volume of input, reactor volume, volume of output) and reaction parameters (for MA and MMA , the equivalent between monomer, 1-dodecanthiol and BAPO is 531.82:1:5, and for Sty the ratio is 531.82:10:5) were set manually. Finally, time-sweeping experiments from 1 to 5 minutes of residence time were carried out with a Python script delivering flow rates to individual syringe pumps. The reaction was performed at 30 °C, 40 °C, 50 °C, 60 °C, 70 °C and 80 °C for the FRP of MA (For the FRP of MMA, the reactions were conducted at 60 °C, 70 °C, 80 °C, and 90 °C; and the FRP of Sty were carried out at 50 °C, 60 °C, 70 °C and 80 °C). All the tubing used in the flow reaction is PFA tubing (0.75 mm I.D., 1/16" O.D.). The scheme of the automated polymerization screening platform is given in Figure S2.

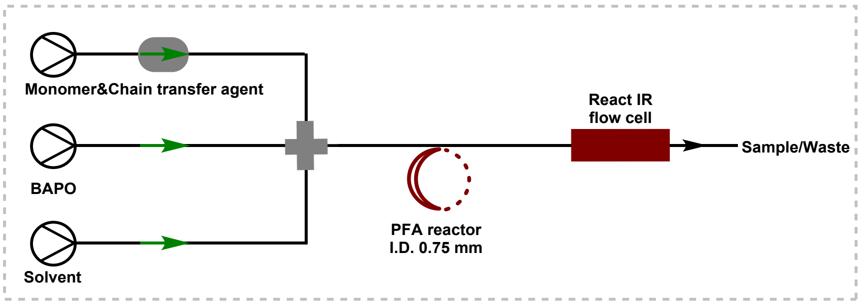

Figure S2. Automated screening platform for time-sweeping experiments of FRP polymerization

Idealized Free Radical Polymerization Kinetics

The rate of initiation is given by:

$$R_{\mathrm{i}} = 2k_{\mathrm{d}}f[I] \quad \text{(S1)}$$

Where k_d is the rate coefficient for the initiator decomposition reaction, [I] is the initiator concentrations, and f is the initiator efficiency. In general, radical generation is assumed to be slower than monomer addition to the initiator fragment radicals. Therefore, the rate of initiation is given by the rate of macroradical generation, as seen in *Eq 1*. After initiation, several additional processes occur in radical polymerization. The first is monomer propagation, leading to chain growth. Propagation occurs with the rate coefficient k_p, where [M] is the monomer concentration and [R•] is the concentration of all growing chains, see Eq. 2.

$$R_p = k_p[\mathrm{M}][\mathrm{R}\bullet] \quad \text{(S2)}$$

In termination, radicals can recombine (or disproportionate), leading to the formation of dead chains. Termination occurs with rate coefficient k_t and the square of the radical concentration. In addition to these fundamental reactions, often classified as so-called ideal radical polymerization, irreversible transfer steps can occur, but these primarily affect chain length rather than kinetics or total radical concentrations as long as reinitiation of the created radical fragments from the transfer

proceeds quickly.[7] As mentioned above, in RAFT, the same initiation, propagation, transfer, and termination processes occur, with an additional step of radical exchange through the RAFT equilibria as noted in Scheme 1, which distribute the propagation probability over all living chains and consequently impose chain length control. [7]

Most polymerizations are initiated by heat. However, photoinitiation is also possible. An advantage of using a photochemical radical initiator over a thermal one is that photoinitiator decomposition is not a function of temperature but of the intensity of incident radiation[8]. Hence, photoinitiation can be decoupled from temperature and is controlled by switching the light on or off, enabling spatiotemporal control over radical generation[6].

Unlike initiation, which can occur either thermally or photochemically, propagation and termination are always thermally controlled processes, following classical Arrhenius laws (see Eq. 3 for the rate of polymerization). It should be noted that the overall rate of polymerization and the rate of propagation are identical in an ideal radical polymerization because the monomer is only consumed during the propagation reaction. In *eq 3*, A_p is the Arrhenius pre-exponential factor for propagation, $E_\mathrm{A}(k_\mathrm{p})$ is the activation energy for propagation, R is the universal gas constant, and T is the absolute temperature. In photoinitiated polymerization systems, an increase in temperature thus has the potential to enhance the propagation rate.[9,10] Consequently, higher temperatures will increase the polymerization rate of these monomer systems.

$$k_p = A_p exp\left[-\frac{E_\mathrm{A}(k_\mathrm{p})}{RT}\right] \qquad \text{(S3)}$$

The IUPAC recommended method for evaluating $E_\mathrm{A}(k_\mathrm{p})$ and k_p for FRP of vinyl monomers is pulsed laser-induced polymerization in conjunction with size exclusion chromatography (PLP-

SEC).[11] Monomers that have been benchmarked with data from across laboratories include methyl methacrylate, styrene, and methyl acrylate [12–14].

In radical processes, termination is unavoidable, as shown in *Scheme 1*, and the rate of termination is given in *eq 4*. Generally, radical termination occurs with diffusion control, whereby several different diffusion processes are rate-determining and depend on the bulk and intrinsic viscosity of the polymerization mixture. As a result, the termination rate coefficient (k_t) is chain length dependent.[15,16] However, a chain length averaged k_t can be used to facilitate analysis of termination kinetics in models that aim to capture key kinetic phenomena.[17,18] An increase in temperature typically has a relatively small yet measurable impact on the termination rate in the radical polymerization of most monomers, including methyl methacrylate, styrene, and methyl acrylate, which is captured by an empirical activation energy of termination ($E_A(k_t)$)[19]

Applying a steady state assumption ($R_i = R_t$) to evaluate free radical concentrations ($[R^\bullet]_{FRP}$) in *eq 5* gives the overall rate of polymerization (R_p, see *eq 6)* without direct measurement of the radical concentration, which is challenging. In photoinitiated polymerization, the radicals are generated by light, and the impact of temperature on termination kinetics is measurable, but typically small compared to propagation and other thermal processes[20]. Nonetheless, when correcting for the contribution of termination, the activation energy for the overall rate of polymerization can be deduced simply by the relationship shown in *eq 7* (see SI for details).

$$R_t = 2k_t[\mathrm{R}\bullet]^2 \qquad \text{(S4)}$$

$$[\mathrm{R}\bullet]_{\mathrm{FRP}} = \sqrt{\frac{k_d f[\mathrm{I}]}{k_t}} \qquad \text{(S5)}$$

$$R_p = k_p[M][\mathrm{R}\bullet] = k_p[M]\sqrt{\frac{k_d f[\mathrm{I}]}{k_t}} \qquad \text{(S6)}$$

$$E_{\mathrm{A}}(R_{\mathrm{p}}) = E_{\mathrm{A}}(k_{\mathrm{p}}) - \frac{1}{2}E_{\mathrm{A}}(k_{\mathrm{t}}) \quad \text{(S7)}$$

In *eq 7*, $E_{\mathrm{A}}(R_{\mathrm{p}})$ is the apparent activation energy of photopolymerization. $E_{\mathrm{A}}(k_{\mathrm{t}})$ is the activation energy of termination and varies slightly between monomers.

Apparent activation energies of Polymerization for Strongly Retarded RAFT Systems

A description of the relationship between temperature and K_{RAFT} relating equilibrium constant to the change in enthalpy (ΔH^{O}) and temperature is as follows:

$$ln\frac{K_2}{K_1} = -\frac{\Delta H^{O}}{R}\left(\frac{1}{T_2} - \frac{1}{T_1}\right) \quad \text{(S8)}$$

Where K_1 and K_2 are equilibrium constant at temperatures T_1 and T_2 respectively,
ΔH^{O} is the standard enthalpy change,
R is the universal gas constant
With higher temperatures favoring the reversal of radical addition to CTA and resulting in less control of molecular weight distributions. In earlier work, the concentration of radicals in a strongly retarded RAFT system, assuming an intermediate termination model is given by

$$[\mathrm{P}\bullet]_{\mathrm{RAFT}} = [\mathrm{P}\bullet]_{\mathrm{FRP}} \times \frac{1}{(1+ K_{\mathrm{RAFT}} \times [\mathrm{CTA}])^{1/2}} \quad \text{(S9)}$$

Where $[\mathrm{P}\bullet]_{\mathrm{FRP}}$ is the radical concentration expected under ideal FRP conditions, K_{RAFT} is the RAFT equilibrium constant, and [CTA] is the chain transfer agent concentration. Substituting into a pseudo first order kinetic law:

$$ln\frac{[\mathrm{M}]_0}{[\mathrm{M}]} = k_{\mathrm{p}}\,[\mathrm{P}\bullet]_{\mathrm{RAFT}}\,t \quad \text{(S10)}$$

In a system with only weak retardation $K_{\mathrm{RAFT}} \times [\mathrm{CTA}] \ll 1$ and hence $[\mathrm{P}\bullet]_{\mathrm{RAFT}} \approx [\mathrm{P}\bullet]_{\mathrm{FRP}}$. Alternatively, in strongly retarded systems $K_{\mathrm{RAFT}} \times [\mathrm{CTA}] \gg 1$, and *eq S10* can be approximated as follows:

$$ln\frac{[M]_0}{[M]} \approx \frac{k_{\mathrm{p}}\,[\mathrm{P}\bullet]_{\mathrm{FRP}}}{(K_{\mathrm{RAFT}} \times [\mathrm{CTA}])^{1/2}}\,t \quad \text{(S11)}$$

Substituting the established radical concentration $[\mathrm{P}\bullet]_{\mathrm{FRP}}$ gives:

$$ln\frac{[M]_0}{[M]}\approx\frac{k_{\mathrm{p}}}{(K_{\mathrm{RAFT}}\times[\mathrm{CTA}])^{1/2}}\sqrt{\frac{k_d f[\mathrm{I}]}{k_t}}t \qquad \text{(S12)}$$

Where k_{d}, [I], f and k_{t} are defined as in the main text. In a photochemical polymerization k_{d}, f, [I] and [CTA] are to a first approximation assumed to be temperature independent. Therefore, the slope of the semilogarithmic plot ($k_{\mathrm{p\text{-}app\text{-}RAFT}}$) is given by:

$$k_{\mathrm{p-app-RAFT}} = \sqrt{\frac{k_d f[\mathrm{I}]}{[\mathrm{CTA}]}}\times k_{\mathrm{p}}\times\sqrt{\frac{1}{K_{\mathrm{RAFT}}\times k_t}} \qquad \text{(S13)}$$

Where the first terms are assumed temperature independent, while k_{p}, K_{RAFT} and k_{t} have some temperature dependence. In general the equilibrium constant can be written in terms of standard enthalpy ($\Delta H^0_{\mathrm{RAFT}}$) and standard entropy ($\Delta S^0_{\mathrm{RAFT}}$)

$$K_{RAFT} = e^{\frac{\Delta S^0_{\mathrm{RAFT}}}{R}}e^{\frac{-\Delta H^0_{\mathrm{RAFT}}}{RT}} \qquad \text{(S14)}$$

Which can be substituted into eq S13 to give

$$k_{\mathrm{p-app-RAFT}} = \sqrt{\frac{k_d f[\mathrm{I}]}{[\mathrm{CTA}]}}e^{\frac{\Delta S^0_{\mathrm{RAFT}}}{R}}k_p\, e^{\frac{\Delta H^0_{\mathrm{RAFT}}}{2RT}}\sqrt{\frac{1}{k_{\mathrm{t}}}} \qquad \text{(S15)}$$

Assuming that k_{p} and k_{t} both have Arrhenius type temperature dependencies with prefactor of A_{p} and A_{t} for propagation and termination respectively and activation energies of $E_{\mathrm{A}}(\mathrm{k_p})$ and $E_{\mathrm{A}}(k_{\mathrm{t}})$ for propagation and termination respectively, eq 15 can be rewritten as:

$$k_{\mathrm{p-app-RAFT}} = A_1 e^{\frac{-\left(E_A(k_{\mathrm{p}})-\frac{\Delta H^0_{\mathrm{RAFT}}}{2}-\frac{E_A(k_{\mathrm{t}})}{2}\right)}{RT}} \qquad \text{(S16)}$$

Where A_1 captures all the temperature independent terms as follows:

$$A_1 = \sqrt{\frac{k_d f[\mathrm{I}]}{[\mathrm{CTA}]}}e^{\frac{\Delta S^0_{\mathrm{RAFT}}}{R}}\frac{A_p}{\sqrt{A_t}} \qquad \text{(S17)}$$

Therefore the rate of polymerization in a strongly retarded RAFT system is given by:

$$R_p = k_p[M][R\bullet] = A_1 e^{\frac{-\left(E_{\mathrm{A}}(k_{\mathrm{p}})-\frac{\Delta H^0_{\mathrm{RAFT}}}{2}-\frac{E_{\mathrm{A}}(k_{\mathrm{t}})}{2}\right)}{RT}}[\mathrm{M}] \qquad \text{(S18)}$$

From this the temperature dependence is given by an Arrhenius type expression with the activation energy given by:

$$E_{\mathrm{A}}(R_{\mathrm{p}}) = E_{\mathrm{A}}(k_{\mathrm{p}}) - \frac{\Delta H^0_{\mathrm{RAFT}}}{2} - \frac{E_{\mathrm{A}}(k_{\mathrm{t}})}{2} \qquad \text{(S19)}$$

While $E_A(k_p)$ and $E_A(k_t)$ are obtainable via PLP experiments[6] and $-\frac{\Delta H^0_{RAFT}}{2}$ is the negative of half the enthalpy change for K_{RAFT} of specific monomer CTA system under consideration. Substituting $E_A(k_p) = 17.3 \pm 0.3$ kJ/mol,[7] $E_A(k_t) = 9 \pm 6$ kJ/mol[6], and using the quantum chemistry estimated ΔH^0_{RAFT} = –95 kJ/mol for acrylate trithiocarbonate (TTC)[8] (monomer, CTA) gives a predicted value of

$$E_A(R_p) = 17.3 + \frac{95}{2} - \frac{9}{2} = 60 \text{ kJ/mol} \quad \text{(S20)}$$

It is important to note the error is a lower bound on the true error as the quantum calculation did not provide an error on the measurement of ΔH^0_{RAFT}, therefore the true error associated with $E_A(R_p)$ will be larger. Using a typical error reported in quantum calculations in the of 10-14 kJ/mol[9], across a range of test systems gives:

$E_A(R_p) \approx 60 \pm 8$ kJ/mol.

Statistical Analysis of Arrhenius Plots

Table S1: Summary of Arrhenius analysis data, including slope of line of best fit and the associated standard error, R^2, P-Value for significance of the Regression line and estimated E_A and its standard error for each system. Each system analyzed by Ordinary Least Squares regression.

Monomer -System	Slope-Arrhenius Plot	Error-Slope Arrhenius Plot	R2-Arrhenius	P-Value Arrhenius	E_A	Error-E_A
MMA-FRP	-2.509441394	0.227146643	0.9839	0.0081	21	2
Sty-FRP	-2.713294618	0.294959413	0.9769	0.0116	23	3
MA-FRP	-0.911593858	0.081460319	0.9690	0.0004	7.6	0.7
MMA-RAFT	-3.009924521	0.0435882	0.9998	0.0092	25	0.4
Sty-RAFT	-4.488496199	0.094774485	0.9996	0.0134	37	0.8
MA-RAFT	-5.676946013	0.504766974	0.9844	0.0078	47	4

In all cases the regression analysis gave R^2 values greater than 0.95, with a very low P-Value (P-Value<<0.05) for the Null hypothesis (no regression relationship). This provides strong support for the regression analysis used in the Arrhenius analysis and the estimated activation energies.

Units of Quantities Subjected to Logarithmic Operations

Several measured parameters are subjected to logarithms during analysis. Notably the semi-logarithmic plot where the ratio of the initial monomer concentration to the monomer

concentration at a later point in time is subject to the natural logarithm, which is equivalent to the negative logarithm of 1-conversion as seen below in eq S21:

$$\ln\left(\frac{[\mathrm{M}]_0}{[\mathrm{M}]_t}\right) = -\ln\left(1 - \mathrm{conversion}\right) \qquad \text{(S21)}$$

In the case of the semilogarithmic plot, the argument of the logarithmic function is explicitly a ratio of two numbers, or a dimensionless number of conversion, and therefore itself dimensionless.

When performing Arrhenius analysis the natural logarithm of the rate coefficient (*k*) or rate of polymerization (*R*). Following the convention established in the literature the resulting ln(k) or ln(R) is dimensionless as it is taken to be the ratio of the measured rate coefficient or reaction rate to the unit of the measurement as follows:[10,11]

$$\ln(k_{1-\mathrm{order}}) = \ln\left(\frac{k_{1-\mathrm{order}}}{1\ \mathrm{s}^{-1}}\right) = \ln(k_{1-\mathrm{order}}) - \ln\left(1\ \mathrm{s}^{-1}\right) \qquad \text{(S22)}$$

$$\ln(k_{2-\mathrm{order}}) = \ln\left(\frac{k_{2-\mathrm{order}}}{1\ \mathrm{M}^{-1}\mathrm{s}^{-1}}\right) = \ln(k_{2-\mathrm{order}}) - \ln\left(1\ \mathrm{M}^{-1}\mathrm{s}^{-1}\right) \qquad \text{(S23)}$$

$$\ln(R) = \ln\left(\frac{R}{1\ \mathrm{M\ s}^{-1}}\right) = \ln(k) - \ln\left(1\ \mathrm{M\ s}^{-1}\right) \qquad \text{(S24)}$$

Where the first example is for a first order reaction rate coefficient, the second is for a second order rate coefficient and the third example is for a rate of reaction. In all cases $\ln(1\ \mathrm{s}^{-1}) = \ln(1\ \mathrm{M}^{-1}\mathrm{s}^{-1}) = \ln(1\ \mathrm{M\ s}^{-1}) = 0$.

References

(1) Haven, J. J.; De Neve, J. A.; Junkers, T. Versatile Approach for the Synthesis of Sequence-Defined Monodisperse 18-and 20-Mer Oligoacrylates. *ACS Macro Lett* **2017**, *6* (7), 743–747.

(2) Reeves, J. A.; Allegrezza, M. L.; Konkolewicz, D. Rise and Fall: Poly (Phenyl Vinyl Ketone) Photopolymerization and Photodegradation under Visible and UV Radiation. *Macromol Rapid Commun* **2017**, *38* (13), 1600623.

(3) Carmona, P.; Moreno, J. The Infrared Spectra and Structure of Methyl Acrylate. *J Mol Struct* **1982**, *82* (3–4), 177–185.

(4) Bradford, K. G. E.; Gilbert, R. D.; Weerasinghe, M. A. S. N.; Harrisson, S.; Konkolewicz, D. Spontaneous Gradients by ATRP and RAFT: Interchangeable Polymerization Methods? *Macromolecules* **2023**.

(5) Plucinski, A.; Willersinn, J.; Lira, R. B.; Dimova, R.; Schmidt, B. V. K. J. Aggregation and Crosslinking of Poly (N, N-dimethylacrylamide)-b-pullulan Double Hydrophilic Block Copolymers. *Macromol Chem Phys* **2020**, *221* (13), 2000053.

(6) Beuermann, S.; Buback, M. Rate Coefficients of Free-Radical Polymerization Deduced from Pulsed Laser Experiments. *Prog Polym Sci* **2002**, *27* (2), 191–254.

(7) Barner-Kowollik, C.; Beuermann, S.; Buback, M.; Castignolles, P.; Charleux, B.; Coote, M. L.; Hutchinson, R. A.; Junkers, T.; Lacík, I.; Russell, G. T. Critically Evaluated Rate Coefficients in Radical Polymerization–7. Secondary-Radical Propagation Rate Coefficients for Methyl Acrylate in the Bulk. *Polym Chem* **2014**, *5* (1), 204–212.

(8) Lin, C. Y.; Coote, M. L. How Well Can Theory Predict Addition–Fragmentation Equilibrium Constants in RAFT Polymerization? *Aust J Chem* **2009**, *62* (11), 1479–1483.

(9) Ghahremanpour, M. M.; Van Maaren, P. J.; Ditz, J. C.; Lindh, R.; Van der Spoel, D. Large-Scale Calculations of Gas Phase Thermochemistry: Enthalpy of Formation, Standard Entropy, and Heat Capacity. *J Chem Phys* **2016**, *145* (11).
(10) Matta, C. F.; Massa, L.; Gubskaya, A. V; Knoll, E. Can One Take the Logarithm or the Sine of a Dimensioned Quantity or a Unit? Dimensional Analysis Involving Transcendental Functions. *J Chem Educ* **2011**, *88* (1), 67–70. https://doi.org/10.1021/ed1000476.
(11) Mills, I. M. Dimensions of Logarithmic Quantities. *J Chem Educ* **1995**, *72* (10), 954. https://doi.org/10.1021/ed072p954.
